# “Children will Love Like You Do”: How Adolescents’ Relationships with Parents Predict the Quality of Best Friendships and Romantic Relations

**DOI:** 10.1007/s10964-025-02172-1

**Published:** 2025-03-28

**Authors:** Loes van Rijn – van Gelderen, Susanne Schulz, Naomi Neervoort, Susan Branje, Geertjan Overbeek

**Affiliations:** 1 Research Institute Child Development of Education, University of Amsterdam, Amsterdam, The Netherlands; 2 Elementary school de Mijlpaal, Amsterdam, the Netherlands; 3 Department Youth and Family, Utrecht University, Utrecht, The Netherlands

**Keywords:** Longitudinal, Adolescence, Parent-adolescent relationship, Best friendships, Romantic relations

## Abstract

Despite valuable insights into the predictors of high-quality romantic relationships, research has yet to examine the mediating role of peer relationship characteristics in linking parent-adolescent relationship quality to romantic relationship quality. In two longitudinal studies, the present research tested whether adolescents’ relationship quality with best friends mediates the associations between relationship quality with mothers or fathers and relationship quality with romantic partners. In Study 1, 164 adolescents in early to middle adolescence (*M*age = 13.76, SD = 0.87; 64% female; 91.5% Dutch ethnical background; 87.4% from intact families) who were in a romantic relationship participated. The sample of Study 2 consisted of 272 adolescents in late adolescence (Mage = 17.23, SD = 0.64; 56% female, 98% Dutch ethical background; 81% from intact families) in a romantic relationship. In both studies, adolescents completed questionnaires about their relationship with their parents at T_1_, their best friends one year later (T_2_), and their romantic relationship another year later (T_3_). The results of Study 1 showed that perceived best friend-adolescent relationship quality at T_2_ was related to romantic relationship quality at T_3_. No other significant longitudinal associations were found. The results of Study 2 (*n* = 272, *M*age = 17.23) showed that perceived relationship quality with mothers and fathers was related to perceived romantic relationship quality at T_3_, and best friends’ relationship quality at T_2_ partially mediated these associations. Overall, the results show that especially during late adolescence, when adolescents have a warm and supportive relationship with their parents, they are more likely to form good quality relationships with peers and, ultimately, intimate and committed romantic relationships.

## Introduction

*“Fathers be good to your daughters, daughters will love like you do*.

*Girls become lovers who turn into mothers. So mothers be good to your daughters too…”*

— John Mayer, “Daughters”

In his award-winning song, using daughters as an example, John Mayer sings about the importance of parents for shaping their children’s intimate relationships later in life. This idea aligns with developmental theories, which emphasize that learning to develop and maintain intimate relationships is a crucial developmental task for adolescents and young adults (Erikson, 1968). Not fulfilling this developmental task can have consequences for psychosocial adjustment, as the quality of romantic relationships plays a key role in well-being, influencing adolescent psychological adjustment (e.g., Aviléz et al., 2021) and long-term health (Loving & Slatcher, 2013). Given these significant effects, understanding the key predictors of romantic relationship quality is essential. Adolescence is a critical developmental period for studying these predictors, as it marks the stage where individuals first engage in romantic relationships (Gonzalez Avilés et al., 2021). Additionally, this period is distinctive due to a qualitative shift in parent-child interactions, making them more equal and reciprocal, and therefore more similar to peer and romantic relationships (Branje, 2018), while peer relationships become increasingly significant sources of support (Bagwell & Bukowski, 2018). Although prior research suggests that peer relationships play a significant part in the connection between parent-adolescent relationships and adolescents romantic relationships (e.g., Furman, 1999), this relation remains unexplored. The current study investigates whether peer relationship quality mediates the association between parent-child relationships quality and subsequent romantic relationships in adolescence.

### Romantic Relationship Quality: The Role of the Parent-Adolescent Relationship

Different theories and perspectives, such as attachment theory (Bowlby, 2005), social-cognitive theory (Bandura, 1986), parenting styles theory (Baumrind, 1966), and social systems theory (e.g., Hartup, 1979), propose that experiences in the family context place children on specific trajectories of social-emotional development (Conger et al., 2001). These theories all assume that children form an internal model based on the interaction with parents that guides their social behavior across diverse social contexts (Burks & Parke, 1996). If experiences were negative, in later life, when youth become involved in romantic relationships, such an internal model surfaces to predispose towards negative expectations and interpretations of the romantic partner’s behavior and intentions, and increase the risk of low relationship satisfaction and relationship break-up. If experiences with parents were positive, such internal models will be supportive and predispose to positive expectations and interpretations of the romantic partner’s behavior and intentions. Additionally, according to social learning theory (Bandura, 1977), individuals observe and imitate behaviors from others, suggesting that children learn and behave based on the observation of the behavior of their parents. Thus, based on these different theoretical models, one can expect continuity in the quality of relationships with partners and others outside the family, including later romantic partners.

Prospective-longitudinal studies found evidence for these theoretical propositions by showing parent-to-partner linkages over time (e.g., Hadiwijaya et al., 2021). Several recent data syntheses have mirrored these findings. A systematic review of 40 studies examining dyadic outcomes in romantic relationships found that attachment security and high parent-child interaction quality predicted better romantic relationship adjustment and more positive observed romantic interactions (River et al., 2022). Also, a meta-analysis of 81 longitudinal studies found that supportive and negative parent-adolescent relationships predicted later supportive and negative qualities of romantic relationships, respectively (Schulz et al., 2022).

### Stepping Stones Towards Love: The Role of Relationship Quality with Best Friends

The studies cited earlier have yielded valuable insights into the potential sources of high-quality romantic relationships. However, they have not examined the role of peer relationships in mediating these effects, despite indications that peer relationships play a significant part in the connection between parent-adolescent relationships and romantic relationships. Compared with the parent-adolescent relationship, relationships with peers, like romantic relationships, usually take shape on a more equal instead of hierarchical basis, with each person having roughly equal status and power (Furman & Shomaker, 2008). Indeed, other studies indicated that friendships may serve as a training ground for the development of “peer affiliative skills” that are conducive to healthy functioning in romantic relationships (e.g., Furman, 1999). Given this unique developmental function of friendships, it can be argued that friendships function as intermediaries in the link between the parent-child bond and subsequent romantic relationships. It may be that warm and supportive parent-child relationships lead to more positive and intimate best friendship experiences and, via these, to the development of more satisfactory—supportive, committed—romantic relationships.

The first evidence for this idea comes from various studies finding associations between the relationship quality with parents, peers, and romantic partners. Concerning the relation between parent and peers, one meta-analysis discovered that early attachment between parents and children is related to the quality of later friendships with peers (Pallini et al., 2014). Another recent longitudinal meta-analysis similarly found that both supportive and negative parent-adolescent relationships predicted future supportive and negative peer relationships, above and beyond current associations (Schulz et al., 2022). Regarding the linkage between peer and romantic relationships, in addition to longitudinal empirical studies (e.g., Roisman et al., 2009), a meta-analysis (Kochendorfer & Kerns, 2020) tested such linkages between positive (global positive quality, support only, intimacy, security) and negative (global negative quality, negative interactions, conflict) qualities between adolescents’ relationships with friends and their romantic relationships, and found small-to-medium positive associations (Kochendorfer & Kerns, 2020). Thus, the body of research so far indicates that the quality of both parent-adolescent relationships and friendships is related to quality of later romantic relationships and suggests that a developmental “stepping stone sequence” towards romantic outcomes may hold true. However, only one study has tested such a developmental sequence from parents through peers toward romantic partners. This study analyzed observational data from adults aged 26–28 who were followed from age 11 onwards, showing that disruptive parenting at age 11 predicted antisocial behaviors and deviancy training later in adolescence, which, in turn, predicted coercion within intimate relationships in adulthood (Ha et al., 2019).

The study provided an initial overview of the developmental sequence as proposed, but its exclusive focus on only one of the dimensions of social relationships (i.e. negative interaction) does not yield a broad insight into the mediating role of peers in the relation between parent-adolescent relationship quality and later adolescent romantic relationship quality. It is important to also consider other, key positive dimensions of social relationships during adolescence, like receiving support, that promote the development of positive relationship patterns and caring and helpful attitudes (Eisenberg et al., 2015), which are suggested to be relevant for relationship quality with peers and romantic partners too.

Also, the number of years between data waves was relatively large. A longer time between assessments may lead to changes in the parent-adolescent relationship or create additional continuous pathways that reduce the link between parent-adolescent relationships and future peer relationships (Fraley & Roisman, 2015). The function of romantic relationships also changes during this period. For early adolescents, having a romantic relationship may primarily confer social status and facilitate fitting in with peers (Overbeek et al., 2003). These earlier romances are still strongly embedded in the broader peer network and “peer affiliative system”—an organized set of behaviors that meet the need of being sociable with peers. Over time, as romantic relationships become more enduring and committed, romantic partners become central figures of support and intimacy (e.g., Furman, 1999), and closeness and attachment motives become the main drivers of adolescent romantic involvement. Due to these developmental differences, the connections between parents, peers, and romantic relationships may structurally change and vary by age, which makes it important to study these connections within shorter periods and across different ages.

## Current Study

While previous research has provided important findings about the predictors of high quality romantic relationships, the role of peer relationship quality as a mediator between the quality of parent-adolescent and romantic relationships remains unexplored. The present research features two three-wave longitudinal studies, using data from 164 adolescents aged 12–16 (Study 1) and 272 adolescents aged 15–22 (Study 2). In both studies, romantically committed adolescents were examined to test whether adolescents’ relationship quality with best friends at T_2_ mediated the associations between relationship quality with mothers or fathers at T_1_ and relationship quality with romantic partners at T_3_. Including both adolescents from early to middle and late adolescence allowed to examine the changing function of romantic relationships. Due to these differences, the connections between parents, peers, and romantic relationships may vary by age, with stronger associations between parent-adolescent relationship quality and romantic relationship quality at older ages.

## Study 1

### Methods

#### Sample

Participating adolescents were part of the Social Development of Adolescents (SODA) study (Overbeek et al., 2010). This study started in 2005 (T_1_), the second wave of data collection (T_2_) took place in 2006, and the third wave of data collection (T_3_) in 2007, and the three-wave longitudinal sample consisted of 774 adolescents. For the current study, only those adolescents who reported about their romantic relationship at T_3_ (*n* = 164) were selected since the aim of the study was to predict romantic relationship quality.

At T_1_, these adolescents were 12–16 years old (*M*age = 13.76, *SD* = 0.87), most of them were female (64%), had a Dutch cultural background (91.5%), and were from intact two-parent families (87.4%). Most adolescents participated secondary vocational education (61%) and the remaining in theoretical and pre-university education (39%). The majority of the adolescents reported that they had one (44.8%) or two (27.6%) brothers/sisters. Most mothers (94.5%) and fathers (93.3%) were born in the Netherlands.

The background characteristics and the quality of the relationships with parents and peers were examined to assess whether the analytical sample differed from the original sample. The analyses revealed no significant differences in most variables between the adolescents included in this study and those excluded. However, it was found that a larger proportion of the adolescents in romantic relationships at T_3_ were girls (*p* < 0.001) and that a higher percentage were enrolled in pre-vocational secondary education (*p* = 0.017).

#### Procedure

The sample was attained by using a stratified sampling procedure. First, 28 secondary schools in and around the city of Nijmegen (the Netherlands) were selected and approached. Twenty-three schools agreed to participate in the study (82%). From January to March 2005, undergraduate students administered questionnaires to the adolescents during a regular lesson (45–50 min) at school. All adolescents and their parents received information about the study, and all provided consent for participation. The IRB of the Radboud University of Nijmegen approved the study. After finishing the data wave, research reports with anonymized group-level information about the social development of the participating adolescents were sent to thank schools for participating. For the T_2_ and T_3_ data collection, the same adolescents were approached again, and the data were collected in the same manner.

### Measures

#### Perceived Parent-Adolescent Relationship Quality

At T_1_, perceived quality of the relationship between parents and adolescents was measured by asking adolescent to complete the Inventory of Parent and Peer Attachment (IPPA; Armsden & Greenberg, 1987), which contains 24 items about the quality of their relationship with their mother (12 items) and father (12 items) (example items: “I am angry with my mother” and “I tell my father about my problems and concerns”). Responses were given on a six-point scale ranging from 1 (never) to 6 (always). A mean score was calculated for mothers and fathers separately. High scores indicate a positive perception of the parent-child relationship. Previous research has shown ample evidence for reliability and construct validity of this measure (e.g., Armsden & Greenberg, 1987). In this study, Cronbach’s alpha was 0.84 for the mother-adolescent relationship scale and 0.86 for the father-adolescent relationship scale.

#### Perceived Best Friend-Adolescent Relationship Quality

At T_2_, adolescents completed the satisfaction and commitment subscales from the Investment Model Scale (IMS; Rusbult et al., 1998), which contains eight items about the quality of the best friendship (example item: “I am satisfied with the relationship with my best friend”). Responses were given on a five-point scale ranging from 1 (not at all true) to 5 (completely true). High scores indicate a positive perception of the relationship with one’s best friend. Previous research has shown strong evidence for reliability and construct validity of this measure (Rusbult et al., 1998). In the present study, Cronbach’s alpha (T_2_) was 0.83.

#### Perceived Romantic Relationship Quality

At T_3_, adolescents completed an adolescence-version of the Triangular Love Scale (TLS; Overbeek et al., 2007), which contains 19 items about the perceived quality of the relationship with one’s romantic partner (example items: “I can tell _______ everything” and “I would rather be with ______ than with someone else”). Answer options ranged from 1 (not at all true) to 7 (completely true), with high scores indicating a positive perception of the romantic relationship. Previous research has shown strong evidence for this measure’s reliability and construct validity (Overbeek et al., 2007). In this study Cronbach’s alpha (T_3_) was 0.92.

### Analyses

Structural Equation Modeling (SEM) was used to test whether perceived best friendship quality at T_2_ mediated the associations between perceived parent-adolescent relationship quality at T_1_, separately for mothers and fathers, and perceived romantic relationship quality at T_3_ while controlling for adolescent age. The package lavaan (Rosseel, 2012) for the software program R 4.2.2 was used to fit the proposed path model to the data. Due to the instability of romantic relationships in adolescence, this study only focused on romantic relationships at T_3_ instead of using a full recursive mediation model with all constructs assessed at all waves. Missing data on relationship quality with parents and peers were handled using full information maximum likelihood. Model fit was determined by the comparative fit index (CFI), the root mean squared error of approximation (RMSEA), and the Standardized Root Mean Square Residual (SRMR). Model fit was deemed good using the following cutoffs: CFI > 0.95, SRMR < 0.08, and RMSEA < 0.06 (Hu & Bentler, 1999). Correlated residuals > 0.10 were deemed significant and subsequently incorporated into the model.

Before running the analyses, the sample size-to-parameters ratio were checked to analyze power. Based on the sample size-to-parameters ratio rule of a minimum N:q ratio of 10:1 and an ideal ratio of 20:1 to establish adequate power (Jackson, 2003), the current N:q ratio of 23:1 for the main analysis was appropriate.

### Results

Missing data ranged from 3.0–3.7% for relationship quality with mothers and relationship quality with fathers, respectively, as well as 18.3% for relationship quality with friends. Little’s missing completely at random (MCAR) test detected no systematic patterns of missingness, χ^2^ = 11.68(6), *p* = 0.070, across study variables. Skewness, kurtosis, and VIF values were below |1| and plot inspections yielded no evidence for violations against normality or linearity assumptions.

On average, adolescents reported that they perceived all their relationships, with parents, best friends, and romantic partners, as of high quality. These positive evaluations did not significantly differ for boys and girls, *F* (4, 124) = 2.25, *p* = 0.068 (see Table 1). Adolescents did report more positively about their relationship with their mothers than with their fathers, *t* (156) = −3.56, *p* < 0.001. Pearson correlations (see Table 2) showed that adolescents’ reports of the quality of their relationship with mothers and fathers and their reports of the quality of their relationship with best friends and romantic partners were positively associated with a small to moderate effect size. Adolescents who held more positive perceptions of the relationship with their mothers also held more positive perceptions of the relationship with their fathers. Furthermore, those who reported positive about their relationship with their best friends also were more positive about their relationship with their romantic partners. No significant correlations between the quality of the relationship with mothers or fathers and the quality of the relationship with peers or romantic relationships were found.

**Table 1:** Means and standard deviations study 1

	Total	Gender^a^	
		Girls	Boys
Perceived parent-adolescent relationship quality T_1_			
Mother-Adolescent relationship	4.48 (0.72)	4.48 (0.67)	4.48 (0.82)
Father-Adolescent relationship	4.23 (0.79)	4.12 (0.76)	4.43 (0.81)
Perceived best friend – adolescent relationship quality T_2_	4.39 (0.51)	4.47 (0.51)	4.25 (0.49)
Perceived romantic relationship quality T_3_	5.43 (0.84)	5.50 (0.83)	5.30 (0.86)

^**a**^A one-way multivariate analysis of variance (MANOVA) showed no statistical differences between adolescent girls and adolescent boys in any of the variables, *F* (4, 124) = 2.25, *p* = 0.068

**Table 2:** Pearson correlations study 1

	1	2	3	4
1. Perceived quality mother-adolescent relationship T_1_	–			
2. Perceived quality father-adolescent relationship T_1_	0.42^**^	–		
3. Perceived quality best friendship T_2_	0.14	0.16	–	
4. Perceived quality romantic relationship T_3_	0.14	0.08	0.20*	–

* *p* < 0.05, ** *p* < 0.01 (2-tailed)

**Fig. 1:**
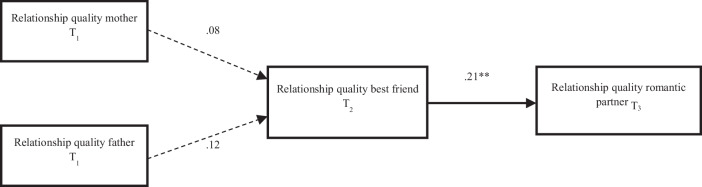
Structural Equal Model Analysis Outcomes Study 1. Note Indirect association from the relationship quality with mothers at T_1_ to later romantic relationship quality at T3 via best friendship quality at T_2_: β_indirect_ = 0.02, *p* = 0.442; Indirect association from the relationship quality with fathers at T_1_ to later romantic relationship quality at T3 via best friendship quality at T_2_: β_indirect_ = 0.03, *p* = 0.280; Indirect association from the relationship quality of both parents together at T_1_ to later romantic relationship quality at T3 via best friendship quality at T_2_: β_indirect_total_ = 0.04, *p* = 0.122. At T_1_, adolescents were, on average, 13.76 years old (*SD* = 0.87. With a one-year gap between data waves, adolescents were one year older at T_2_ and two years older at T_3_. Note ** *p* < 0.01 (2-tailed)

The full mediation model fit the data well, χ^2^(2) = 1.79, *p* = 0.410, RMSEA < 0.001, CFI > 0.999, SRMR = 0.26, and explained a small proportion of variance in the quality of romantic relationships at T3 (*R*^2^ = 0.06). Adding direct effects of relationship quality with mothers and fathers at T_1_ predicting relationship quality with romantic partners at T_3_ did not improve model fit, Δχ^2^(2) = 1.79, *p* = 0.410, suggesting that the more parsimonious full mediation model should be retained. Adolescents’ perceptions of their relationship quality with their mother (β = 0.08, *p* = 0.420) or father (β = 0.12, *p* = 0.226) did not significantly predict their perceptions of relationship quality with their best friend one year later (see Fig. 1). However, when adolescents reported their relationship with their best friend to be of higher quality, they also reported higher quality romantic relationships one year later (β = 0.21, *p* = 0.014). No indirect associations were found from relationship quality with mothers (β_indirect_ = 0.02, *p* = 0.442), fathers (β_indirect_ = 0.03, *p* = 0.280), or both parents together (β_indirect_total_ = 0.04, *p* = 0.122) at T_1_ to romantic relationship quality at T_3_ via best friendship quality at T_2_.

### Sensitivity Analyses

Using the Mahalanobis Distance, two multivariate outliers were identified. Conducting the analysis without outliers resulted in the same conclusions as the analysis with outliers. To check whether the results were the same for adolescent boys and girls, multigroup analyses were run to examine whether adolescent gender moderated the associations between relationship quality with mothers and fathers, best friends, and romantic partners. Results revealed that the unconstrained model did not significantly differ from the constrained model, Δχ^2^ (9) = 10.97, *p* = 0.278, suggesting that the model did not differ for boys and girls.

## Study 2

### Methods

#### Sample

Adolescents were selected from the ongoing Research on Adolescent Development and Relationship, which consists of two cohorts: RADAR-Young (Branje & Meeus, 2018) and RADAR-Old (Branje, 2024). Data from the RADAR-Young (Waves 5, 6, and 7) and RADAR-Old (Waves 6, 7, and 8) studies were combined to create one new dataset of same-aged adolescents with three data waves. The resulting three-wave (from hereon: T_1_, T_2_, and T_3_) longitudinal sample consisted of 993 adolescents. Again, a backtracking approach was employed by including only adolescents involved in a romantic relationship at T_3_, leading to an analytic sample of 272 adolescents.

At T_1_, adolescents were between 15–22 years old (*M*age = 17.23, *SD* = 0.64), predominantly female (56%), from Dutch cultural background (98%), and from intact two-parent families (81%). Most adolescents participated in theoretical and pre-university education (57%) or secondary vocational education (23%), and the remaining adolescents participated in higher professional education (5%) or were participating in other activities and work (15%). The average age of mothers at T_1_ was 47.88 years (*SD* = 5.26), while the average age of fathers was 50.68 years (*SD* = 5.10). Most adolescents (91.2%) came from families with a middle or high socio-economic status, based on parents’ occupation level.

Adolescents in romantic relationships at T_3_ were similar regarding background variables and the quality of relationships with parents and peers. However, adolescents who reported a romantic relationship at T_3_ were more likely to be female (*p* = 0.002) and to have slightly older best friends at T_2_ (M_diff_ = 0.25 years, *p* = 0.036).

#### Procedure

Sampling for the RADAR-Young study occurred through randomly selected schools in the province of Utrecht and three other large cities in the Netherlands. Of the 429 contacted schools for the RADAR-Young study, 296 (69%) agreed to participate, of which 230 eventually were used for sample procedures. Of all eligible families within these schools (*n* = 1081), 687 agreed to participate. For the RADAR-Old study, families were recruited from a larger longitudinal cohort study, resulting in a sample of 306 adolescents.

Adolescents completed questionnaires during annual home visits, with 1-year time intervals. Adolescents received monetary compensation (20 euro’s for RADAR-Young, 10 euro’s for RADAR-Old) for their participation in each of the home visits. Adolescents and their parents both provided consent for participation in the study, which was approved by the Medical-Ethical Committee (METC) and the Faculty Ethical Review Board of Utrecht University.

### Measures

#### Perceived Quality of Relationships

Perceived mother-adolescent relationship quality (T_1_), perceived father-adolescent relationship quality (T_1_), perceived best friendship quality (T_2_), and perceived romantic relationship quality (T_3_) were measured with the short version of the Network of Relationships Inventory (NRI; Furman & Buhrmester, 2009). This instrument contains eight items about support and six items about negative interactions for each of the relationships. An example item of the support scale is “*How much do you really care about your father/mother/best friend/romantic partner?*”. An example from the negative interactions scale is “*Do you and your father/mother/best friend/romantic partner get on each other’s nerves?*”. Responses were given on a five-point scale ranging from 1(a little or not at all) to 5 (more is not possible). The support and negative interaction scales were averaged per relationship so that high scores indicate the overall quality of the relationship. Previous research has shown strong evidence for the reliability and construct validity of the NRI (De Goede et al., 2009). The combined relationship quality scales showed acceptable internal consistency in the relationship with mothers (α = 0.80), fathers (α = 0.71), best friends (α = 0.72), and romantic partners (α = 0.73).

#### Analyses

For this study, the same SEM as in Study 1 was conducted to answer the research questions. Again, the sample size-to-parameters ratio to see was checked to see if the study had enough power. The N:q ratio was 38:1, and because an N:q ratio of 20:1 is considered a sufficient ratio to establish adequate power (Jackson, 2003), this was appropriate.

### Results

Missing data ranged from 5.1–7.0% for relationship quality with mothers and relationship quality with fathers, respectively, as well as 7.7% for relationship quality with friends. Little’s missing completely at random (MCAR) test detected no systematic patterns of missingness, χ^2^ = 9.70(6), *p* = 0.138, across variables. Skewness, kurtosis, and VIF values were below |1| and plot inspections yielded no evidence for violations against normality or linearity assumptions.

Similar to the adolescents from Study 1, the adolescents in this study described their relationships with parents, best friends, and romantic parents as of high quality (see Table 3). However, girls reported more positively about their relationship with their best friends than boys did, *F* (1, 234) = 14.55, *p* = < 0.001, and adolescents reported more positively about their relationship with their mothers than with their fathers, *t* (252) = 4.80, *p* < 0.001. Pearson correlations (see Table 4) showed that all bivariate relationships were positive and moderately strong: adolescents who had positive perceptions of one of the four relationships also had positive perceptions of their other relationships.

**Table 3:** Means and standard deviations study 2

	Total	Gender^a^	
		Girls	Boys
Perceived parent-adolescent relationship quality T_1_			
Mother-Adolescent relationship	4.05 (0.48)	4.07 (0.49)	4.02 (0.46)
Father-Adolescent relationship	3.87 (0.58)	3.86 (0.59)	3.88 (0.56)
Perceived best friend – adolescent relationship quality T_2_	4.06 (0.45)	4.16 (0.41)	3.94 (0.46)
Perceived romantic relationship quality T_3_	4.29 (0.41)	4.32 (0.40)	4.25 (0.42)

^a^A one-way multivariate analysis of variance (MANOVA) showed statistical differences between girls and boys, *F* (4, 231) = 4.41, *p* = 0.002. Post-hoc analyses revealed that girls were more positive about the relationship with their best friend than boys were, *F* (1, 234) = 14.55, *p* = < 0.001

**Table 4:** Pearson correlations study 2

	1	2	3	4
1. Perceived quality mother-adolescent relationship T_1_	–			
2. Perceived quality father-adolescent relationship T_1_	0.39^**^	–		
3. Perceived quality best friendship T_2_	0.27^**^	0.30^**^	–	
4. Perceived quality romantic relationship T_3_	0.32^**^	0.30^**^	0.26^**^	–

** *p* < 0.01 (2-tailed)

**Fig. 2:**
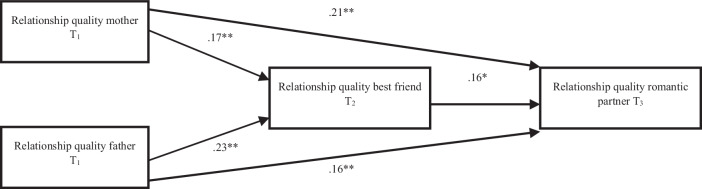
Structural Equal Model Analysis Outcomes Study 2. Note Indirect association from the relationship quality with mothers at T_1_ to later romantic relationship quality at T3 via best friendship quality at T_2_: β_indirect_ = 0.03, *p* = 0.073; Indirect association from the relationship quality with fathers at T_1_ to later romantic relationship quality at T3 via best friendship quality at T_2_: β_indirect_ = 0.04, *p* = 0.040; Indirect association from the relationship quality of both parents together at T_1_ to later romantic relationship quality at T3 via best friendship quality at T_2_: β_indirect_total_ = 0.07, *p* = 0.020. At T_1_, adolescents were, on average, 17.23 years old (*SD* = 0.64), at T_2_ 18.23 years old (*SD* = 0.64), and at T_3_ 19.79 (*SD* = 0.54) years old. Note * *p* < 0.05, ** *p* < 0.01 (2-tailed)

The full mediation model failed to provide a good fit, χ^2^(2) = 38.81, *p* < 0.001, RMSEA = 0.179, CFI > 0.575, SRMR = 0.066. Model inspection revealed substantial correlation residuals for the direct effects from relationship quality with mother (0.24) and father (0.20) to relationship quality with romantic partner, which were thus added to the model. The partial mediation model significantly improved model fit, Δχ^2^(2) = 23.391, *p* < 0.001, and explained moderate proportions of variance in the quality of best friendships at T_2_ (*R*^2^ = 0.12) and romantic relationships at T_3_ (*R*^2^ = 0.16). As expected, when adolescents reported higher relationship quality with their mother (β = 0.17, *p* = 0.013) and father (β = 0.23, *p* = 0.001), they also reported higher relationship quality with their best friend one year later (see Fig. 2). In turn, when adolescents reported higher relationship quality with their best friend, they reported higher relationship quality with their romantic partner one year later (β = 0.16, *p* = 0.010). The indirect effect for relationship quality with fathers was significant, indicating that higher relationship quality with fathers (β_indirect_ = 0.04, *p* = 0.040), but not mothers (β_indirect_ = 0.03, *p* = 0.073) predicted higher relationship quality with romantic partners via higher relationship quality with best friends across late adolescence. The total indirect effect for relationship quality with both parents was significant (β_indirect_total_ = 0.07, *p* = 0.020). Relationship quality with mothers (β = 0.21, *p* = 0.001) and fathers (β = 0.16, *p* = 0.012) at T_1_ further directly predicted relationship quality with romantic partners at T_3_.

#### Sensitivity Analyses

Using the Mahalanobis Distance method one multivariate outlier was found. When this outlier was removed, similar analysis outcomes as before were reached, except that the indirect effect for relationship quality with mothers was now significant (β_indirect_ = 0.04, *p* = 0.040) and the indirect effect for relationship quality with fathers was not (β_indirect_ = 0.04, *p* = 0.055). These results for indirect effects were opposite to those for the main analyses, so they should be interpreted with caution. Multigroup analyses revealed that the unconstrained model fit significantly better than the constrained model, Δχ^2^ (11) = 23.58, *p* = 0.015. However, this model failed to provide acceptable fit, χ^2^(4) = 12.48, *p* = 0.014, RMSEA = 0.125, CFI > 0.887, SRMR = 0.024. Stepwise constraining of each path in the model revealed no significant worsening of model fit, suggesting that none of the associations in the model significantly differed for adolescent boys and girls.

## Discussion

Different theories propose that high parent-adolescent relationship quality foretell satisfactory romantic relationship functioning at a later life stage. It was proposed that friendships may act as a mediator between the parent-child relationship and future romantic partnerships, and conducted two three-wave longitudinal studies with adolescents in early to middle (*n* = 164) and late adolescence (*n* = 272) to test this idea. Both studies showed that the quality of the relationship with friends predicted the quality of romantic relationships a year later. In other words, if adolescents had a positive relationship with their peers, they were more likely to evaluate their romantic relationships positively. While preliminary evidence that relationship quality with peers partially mediated the associations between relationship quality with parents and relationship quality with romantic partners in late adolescents (Study 2) was found, this was not of the case for younger adolescents (Study 1).

The finding that the quality of best friendships and romantic relationships were interrelated over time corresponds to the idea that friendships may serve as a training ground for developing social skills with those equal in status (e.g., Furman, 1999) and adds to earlier empirical work on this topic (e.g., Kochendorfer & Kerns, 2020). This current research indicates that the relation between peer relationships and romantic relationships is present in early-to-middle adolescence as well as in late adolescence. Thus, although romantic relationships may develop into more long-term and committed partnerships over time, the quality of these relationships still tends to be related to the quality of earlier peer relationships.

In contrast to the results of a recent meta-analysis (Schulz et al., 2022), the current study did not find support for the idea that the perceived quality of the mother-adolescent or father-adolescent relationship was related to the quality of the best friend or romantic relationship in early to middle adolescence. It may be the case that many adolescents in this specific age group have friendships and romantic relationships that are not as close and intimate as their relationships with their parents - limiting the influence of their mental representations of their intimate bond with their parents on their peer relationships. In line with this theoretical notion, it may be only later, when peer relationships become more enduring and committed, with best friends and romantic partners serving as attachment figures (e.g., Furman, 1999), that the quality of the parent-adolescent relationship is related to the quality of friendships and romantic relationships, hence the findings from Study 1.

The findings from Study 2 indeed provided evidence to support the notion that positive parent-adolescent relationships can lead to better quality relationships with best friends and romantic partners in late adolescence by showing that good relationships with parents were associated with high-quality relationships with best friends, which were in turn, associated with high-quality romantic relationships. An explanation for this finding might be that, over time, adolescents develop more mature friendships with an optimal balance of autonomy and connectedness, while at the same time, parent-adolescent relationships become more reciprocal and egalitarian (e.g., Hadiwijaya et al., 2017). Also, romantic relationships become more stable and committed later in life, with romantic partners becoming central figures of support and intimacy (e.g., Furman, 1999). Having more close and durable friendships and romantic relationships in which closeness and attachment motives take precedence might enhance the relevance of working schemes of relationships modeled after the parent-child relationship.

There are some potential alternative explanations of the findings that warrant attention. An earlier meta-analysis showed that more supportive relationships with parents were related to more supportive relationships with peers (and not the other way around). In contrast, negative parent-adolescent relationships were also predicted by the quality of the peer-adolescent relationships (Schulz et al., 2022). In the current study, the parent-peer linkage might also be bidirectional. Additionally, the duration of the relationships was not included in the analyses. Adolescents who have peer and romantic relationships of longer duration may see their relationships with peers and romantic partners meeting more of their emotional and support needs (i.e. they become more central figures of support and intimacy; Furman, 1999), which could make the connections between the quality of the parent-adolescent relationship and the quality of the peer and romantic relationships stronger. Future studies could test this hypothesis by including the duration of the relationship as a moderator.

This study is unique in that it presents results from three-wave longitudinal analyses—across multiple well-powered cohorts of adolescents from the general population—of a core developmental hypothesis regarding individuals’ relational development. Yet, several limitations warrant mentioning. First, in both studies, the data consisted of self-reports. Theoretically, one expects that the perception of adolescents’ current relationships depends on how they *perceived* and *constructed* previous relationships with parents (Overbeek et al., 2007). While it would be interesting to explore how these working models of relationships are rooted in actual relational experiences, it might make less sense to assume that the perception of one’s current relationship depends on how previous relationships were viewed by other reporters or observers. However, the used approach may have led to an inflation of parameter estimations by uni-informant bias. For instance, based on personality traits like agreeableness, one could be inclined to rate all relationships more positively (Branje et al., 2005), which would (partly) explain the longitudinal associations.

Second, different instruments and incentives were used in the two studies, potentially contributing to the dissimilar outcomes. Also, in both studies, it might be that adolescents differed in how close the relationship was with their so-called best friend. As with previous-parent-child relationships, theoretically one could expect that how adolescents perceive “best” friendships would matter – making it less of a problem that no clear definition was provided. However, since previous studies have shown that gender differences in adolescents’ definitions of friendships exist (e.g., Kitts & Leal, 2021), this might have impacted the results. At the same time, the sensitivity analyses did not reveal different results for girls and boys, which makes it less likely that this limitation significantly influenced the results.

Third, although cross-relational continuity was examined, the study did not focus on how this continuity comes about. That is, no full mediation model with all constructs assessed at all waves was tested. Also, purposefully, the study did not examine growth trajectories or controlled for previous levels of dependent variables because the tested stepping stone model does not relate to intra-individual change—it does not have a “slope hypothesis” but only has an “intercept hypothesis.” In non-statistical terms, the model does not specify that when previous experiences with parents were positive, one will expect a (continuous) increase in the perceived quality of one’s romantic relationship during adolescence. Instead, it would be expected that adolescents—compared to their peers—would report relatively high on a measure of romantic satisfaction if they previously had reported very positively about their relationship with their parents—a rank-order phenomenon.

Fourth, the studies did not include many adolescents from different backgrounds, such as from diverse ethnic groups and diverse family types. Adolescent romantic relationships and culture are closely related, and, as such, each adolescent has their own set of cultural expectations when entering a relationship (Coates, 1999). Thus, it is essential to explore whether parents have a similar impact on the quality of romantic relationships in diverse cultures and to ensure the generalization of the findings to a global population. It is possible that attachment processes are similar across cultures, but that the timing of effects might depend on cultural norms about when it is appropriate to engage in romantic relationships. In addition, previous research has indicated that going through a divorce is associated with less favorable romantic outcomes, such as low commitment to romantic relationships (Cui & Fincham, 2010), increased instability (Gachler et al., 2009), and greater likelihood of infidelity and conflict in romantic relationships (Chen et al., 2006). These effects might be partly explained by the impact of parental divorce on the quality of the parent-adolescent relationship (e.g., Van Dijk et al., 2020). Nevertheless, having more adolescents who experienced parental divorce in the sample could have led to more variation in romantic relationship quality, leading to different results.

## Conclusion

Is it true that, as John Mayer sang, children will love like their parents? Various theoretical perspectives support this idea by suggesting that a parent-adolescent relationship of good quality can predict the ability to form satisfying friendships and, consequently, romantic relationships later in life. However, longitudinal studies testing this developmental model across different phases of adolescence were lacking until now. The current set of studies, as a first, demonstrate that when adolescents have a warm and supportive relationship with their parents, they are more likely to form satisfying friendships and, ultimately, intimate and committed romantic relationships in late adolescence—although effect sizes (to be expected predicting complex, multifaceted relational phenotypes) are small. Uncovering this “developmental sequence of love” during adolescence reveals the crucial role of sensitive and supportive parenting. The current research also indicates that the relation between peer relationships and romantic relationships is evident in early-to-middle adolescence as well as in late adolescence. This suggests that helping adolescents build positive relationships with their peers, regardless of the age of the adolescents, can enhance the quality of their romantic relationships later in life.

### Data Sharing

The SODA dataset is not deposited, but syntax and output are available upon request through the first author. The RADAR dataset is available in the DANS repository, 10.17026/dans-zrb-v5wp. Restrictions apply to the availability of these data for ethical and GDPR reasons, and so are not publicly available. However, data are available from the authors upon reasonable request and with permission of the RADAR.
